# Critically Appraised Topics (CATs) in Veterinary Medicine: Applying Evidence in Clinical Practice

**DOI:** 10.3389/fvets.2020.00314

**Published:** 2020-06-26

**Authors:** Marnie L. Brennan, Sebastian P. Arlt, Zoe Belshaw, Louise Buckley, Louise Corah, Hannah Doit, Virginia R. Fajt, Douglas J. C. Grindlay, Heather K. Moberly, Lisa D. Morrow, Jenny Stavisky, Constance White

**Affiliations:** ^1^Centre for Evidence-based Veterinary Medicine, University of Nottingham, Loughborough, United Kingdom; ^2^Clinic for Animal Reproduction, Faculty of Veterinary Medicine, Freie Universität Berlin, Berlin, Germany; ^3^PDSA Nottingham, Nottingham, United Kingdom; ^4^Deanery of Clinical Sciences, College of Medicine & Veterinary Medicine, University of Edinburgh, Edinburgh, United Kingdom; ^5^School of Veterinary Medicine and Science, University of Nottingham, Loughborough, United Kingdom; ^6^Veterinary Physiology and Pharmacology, Texas A&M University College of Veterinary Medicine and Biomedical Sciences, College Station, TX, United States; ^7^Centre of Evidence Based Dermatology, University of Nottingham, Nottingham, United Kingdom; ^8^Medical Sciences Library, University Libraries, Texas A&M University, College Station, TX, United States; ^9^Fremont Veterinary Clinic, Portland, OR, United States

**Keywords:** critically appraised topic (CAT), knowledge summary, BestBETs, evidence synthesis, evidence-based veterinary medicine, veterinary medicine, clinical practice

## Abstract

Critically appraised topics (CATs) are evidence syntheses that provide veterinary professionals with information to rapidly address clinical questions and support the practice of evidence-based veterinary medicine (EBVM). They also have an important role to play in both undergraduate and post-registration education of veterinary professionals, in research and knowledge gap identification, literature scoping, preparing research grants and informing policy. CATs are not without limitations, the primary one relating to the rapid approach used which may lead to selection bias or restrict information identified or retrieved. Furthermore, the narrow focus of CATs may limit applicability of the evidence findings beyond a specific clinical scenario, and infrequently updated CATs may become redundant. Despite these limitations, CATs are fundamental to EBVM in the veterinary profession. Using the example of a dog with osteoarthritis, the five steps involved in creating and applying a CAT to clinical practice are outlined, with an emphasis on clinical relevance and practicalities. Finally, potential future developments for CATs and their role in EBVM, and the education of veterinary professionals are discussed. This review is focused on critically appraised topics (CATs) as a form of evidence synthesis in veterinary medicine. It aims to be a primary guide for veterinarians, from students to clinicians, and for veterinary nurses and technicians (hereafter collectively called veterinary professionals). Additionally, this review provides further information for those with some experience of CATs who would like to better understand the historic context and process, including further detail on more advanced concepts. This more detailed information will appear in pop-out boxes with a double-lined surround to distinguish it from the information core to producing and interpreting CATs, and from the boxes with a single line surround which contain additional resources relevant to the different parts of the review.

## Evidence-Based Veterinary Medicine

Evidence-based veterinary medicine (EBVM) can be defined as the application of scientifically generated evidence into clinical veterinary practice, whilst synergistically incorporating the expertise of the veterinary professional, the specific features of the patient and the values of the owner (1). In order to practice EBVM, it is important for veterinary professionals to keep up to date with the latest research findings to ensure they are providing the best possible care for patients they treat (2). This is challenging due to the vast amount of information published every day, and for professionals working in the current framework of veterinary practice, it is difficult to find the time (3). Additionally, it can be challenging to interpret the published literature to determine whether it is of relevance, to identify whether the results of the studies are valid and the conclusions drawn by the authors appropriate (4). Structured summaries of the published research (evidence syntheses) are of huge benefit to veterinary professionals, allowing them to easily and quickly incorporate evidence into clinical practice.

## Evidence Syntheses: Reviews and Critically Appraised Topics

Most people will have heard of “literature reviews” or “narrative reviews.” They are typically written by experts who summarize a number of information sources, often peer reviewed articles, on a particular area of interest and offer conclusions. They rarely control for bias or follow a specific methodology for identifying and selecting the sources that are included. Without these standards, the review may not cover the topic inclusively and the conclusions may support a specific agenda or view.

Evidence syntheses [also known as “research syntheses” or “knowledge syntheses” (5)] collectively describe a range of approaches for more objectively summarizing the literature (6). Methodological differences between types of evidence syntheses include the processes and standards for identifying, selecting, and analyzing the sources reviewed and included (7). These methodological variations support differences in the efforts to control for bias, size of the project team, comprehensiveness, and duration. Systematic reviews (SRs) are a type of evidence synthesis that follow a structured methodology to ensure all the available evidence (published and unpublished) is identified and considered (8).

Critically appraised topics (CATs) use the principles of SRs to minimize bias in gathering and appraising evidence, but do so much more quickly (5, 9–11). A CAT is based on a question of interest originating from professionals asking the question after an encounter with a particular clinical case or situation (12).

**Figure 1:**
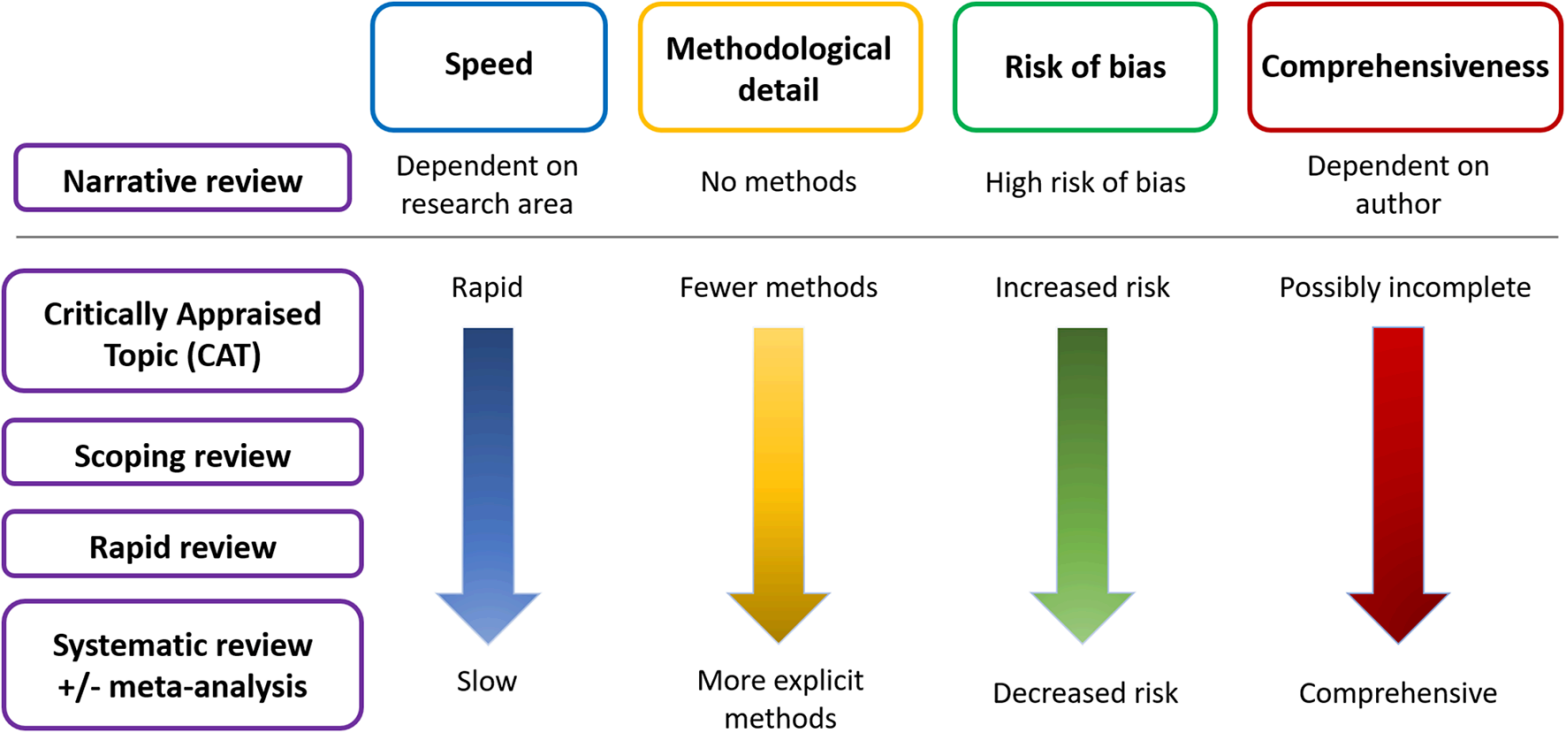
Schematic of the main differences between the types of literature review.

Evidence synthesis methods exist along a spectrum of brevity and detail; CATs are the quickest, SRs the lengthiest and most thorough, and other types fall in between (13). As well as speed and detail, the scope of the question, qualifications of the reviewer and the risk of bias may also differ between the different types of review (3) (Figure 1).

Publications describe the different types of evidence synthesis methods that have been used in research in health related (6, 7), and agri-food public health areas (14). These studies interestingly do not include CATs as a type of review, which may be an oversight, or indicative of why and how they are used.

## Origin of Critically Appraised Topics

The CAT concept was developed by a group of internal medicine fellows at McMaster University, Canada (15) and refined in collaboration with a clinical group at Oxford University in the UK (16). CATs were created so fellows could add value to discussions during case rounds and journal clubs (17). It was felt that for busy clinicians, spending a lot of time trying to keep up to date with the wealth of literature was challenging, and traditional methods of searching and reviewing were not applicable (16). Furthermore, for evidence-based medicine (EBM) to be implemented successfully into clinical practice, access to relevant evidence needed to be quickly and easily accessible at the point of patient care (18). CATs helped clinicians learn the skills to search for relevant evidence, critically appraise and write evidence summaries—fundamental skills to practice and teach evidence-based medicine (17). The first CAT process was published in 1993 (19) with the first peer-reviewed article about CATs published in 1995 (17).

The “quick and dirty” applied approach of a CAT makes it versatile and practical to be translated to other disciplines. Physiotherapy, occupational therapy, dermatology, urology, radiology, nursing (9, 11–13, 20), management (21), and education (22) have embraced the CAT approach. The first mention of veterinary professionals using a CAT format was by Cockcroft and Holmes (2); according to the authors, veterinary CATs did not exist at that time. Soon after, a discussion followed about the role of CATs in veterinary education by Hardin and Robertson (23).

## Uses of the Method in Veterinary Medicine

CATs are primarily used in veterinary clinical practice to answer clinical queries resulting from specific cases or conundrums (13). These could be in relation to the case itself, the clinical professionals' knowledge or familiarity with treatments, diagnostic tests, management regimes or surgical approaches, or questions arising from the client. The CAT methodology has been described as a way of closing the gap between clinical research and clinical decision making (15).

CATs are also used in veterinary undergraduate and post-registration education (9, 24) to investigate a clinical question by teaching searching skills, critical appraisal of scientific literature, and the principles of EBVM (23, 25). This is important as research suggests veterinary clinicians (26), mirroring those in other disciplines (9), do not always use an evidence-based approach (e.g. using peer reviewed publications) when finding literature to aid clinical decision making. This is despite EBVM being increasingly recognized as a core skill for all practitioners. The value of the CAT approach in teaching EBVM and critical appraisal skills has been recognized by a variety of veterinary educators globally (3, 27–29).

Other uses in veterinary medicine for CATs are those relevant to any structured review of the literature, including identification of knowledge/research gaps (24, 30), preparation for research grant applications and for informing policy (14, 31).

## What are the Steps in Undertaking a Critically Appraised Topic?

**Figure 2:**
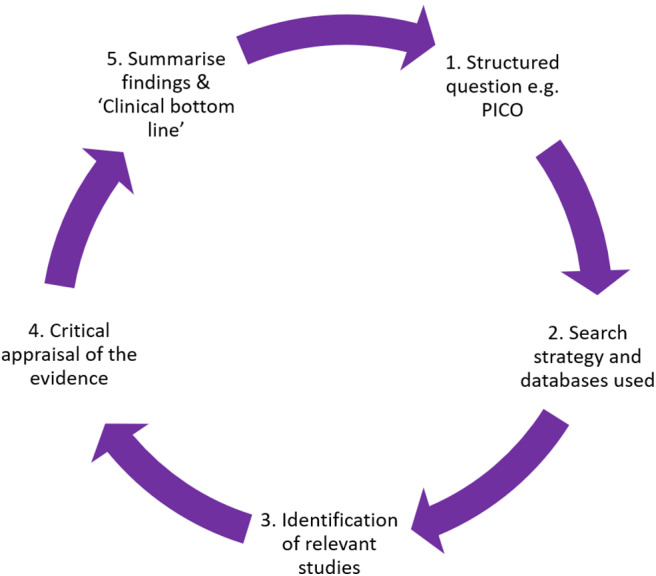
Diagrammatic overview of the CAT process.

For clinicians, it is useful to think of the CAT process in sequential steps or stages (32, 33). A schematic of the CAT process can be seen in Figure 2.

**Figure 3:**
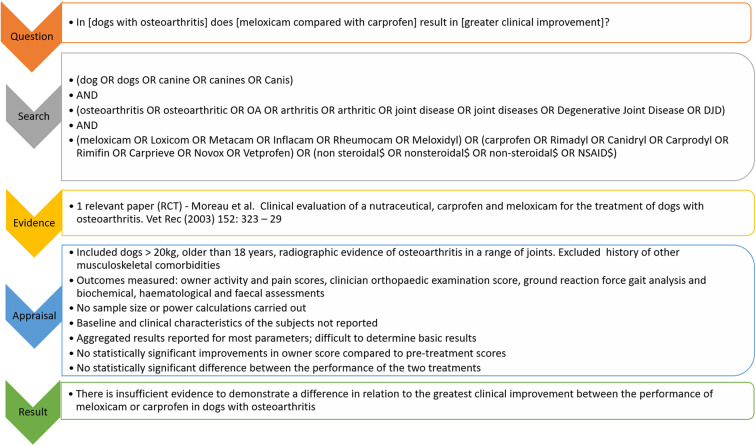
Overview of the example CAT provided on the use of NSAIDs (meloxicam vs. carprofen) in dogs with osteoarthritis.

The CAT process is explained in the steps below, using an example to highlight key points, with an overall summary of the example demonstrated in Figure 3. Additional information for those more experienced in the CAT methodology is provided in the pop out boxes with double-lined surrounds.

### 1. Define CAT Question Using Structured Approaches

Transforming a clinical question into a searchable query can be daunting (34). One of the ways to facilitate this process is by using a defined question format. A PICO question (13), where PICO stands for Patient, Intervention, Comparator, Outcome, are the important components a searching strategy should contain (35) if the question relates to treatment efficacy or interventions (for example drugs, vaccines, or surgical procedures). If the question relates to the accuracy of diagnostic tests, then a slightly different format might be appropriate e.g., PIT—Population, Index Test, Target condition or disease (5). Alternative formats for clinical question including prevalence of disease, etiology and comorbidities are described by O'Connor and Sargeant (5).

The PICO format is often illustrated as:

In [patient group] does [intervention and comparator] result in [outcome]

The following clinical scenario will demonstrate the steps of the CAT process.

You have been treating Miley, a 12-year-old Doberman, for osteoarthritis for the past two years. Her owners bring her in for a check-up. On clinical examination you find further reduction in her range of movement, and some signs of pain when you manipulate both of her hind limbs. She is currently on carprofen. Miley's owner asks about meloxicam, as one of the dogs at the park where he walks Miley receives it for a similar problem. You wonder whether Miley may show a greater improvement in clinical signs if she is treated with meloxicam instead of carprofen.

In this clinical scenario, the PICO question might be:

P = Patient group (dogs with osteoarthritis)

I = Intervention (meloxicam)

C = Comparator (carprofen)

O = Outcome (greatest clinical improvement)

In [dogs with osteoarthritis] does [meloxicam compared with carprofen] result in [greater clinical improvement]?

It is possible that further defining the patient group (e.g. forelimb osteoarthritis vs. osteoarthritis) and outcome (e.g. lameness determined by a visual analog scale vs. general clinical improvement) would permit the evidence to be evaluated for applicability more specifically to the clinical case in front of the veterinary professional.

By converting the scenario to a structured PICO format, a search strategy can be focused to answer the question, and appraisal of the evidence (see section below) can focus on the applicability as it relates to the specific question. For further information about searching see the box entitled “General references for defining a question.”

**General references for defining a question:**

De Brun C, Pearce-Smith N. *Searching Skills Toolkit: Finding the Evidence*. Oxford, UK: Wiley-Blackwell. ISBN: 9781118463130 (2009).

EBVM Learning “Ask” module (http://www.ebvmlearning.org/ask/)

EBVM Toolkit 1 (https://knowledge.rcvs.org.uk/document-library/ebvm-toolkit-1-asking-an-answerable-clinical-question/)

PICOvet website (https://pico.vet/index.html)

### 2. Creating a search strategy

#### Identifying Search Terms

The PICO question can then be used to search for published evidence relating to the clinical scenario it describes. The first step is identifying search terms that will find the greatest number of relevant publications whilst omitting those that are irrelevant (2). Publications may be inconsistent in the terms used in their titles and abstracts to describe the same thing, so creating a list of synonyms for each PICO component will help to ensure that relevant material is located. By being as comprehensive as possible within each of the P, I, C and O components, the greatest amount of relevant material can be identified.

The search terms identified for the example PICO question are shown in Table 1.

**Table 1:** Search terms identified for the PICO question “In [dogs with osteoarthritis] does [meloxicam compared with carprofen] result in [greater clinical improvement]?”

**Patient**		**Intervention**	**Comparator**	**Outcome**
**Species**	**Condition**			
Dog Dogs Canine Canines *Canis*	Osteoarthritis Osteoarthritic OA Arthritis Arthritic Joint disease Joint diseases Degenerative Joint Disease DJD	Meloxicam Loxicom Metacam Inflacam Rheumocam Meloxidyl Non steroidal Nonsteroidal Non-steroidal NSAID NSAIDs	Carprofen Rimadyl Canidryl Carprodyl Rimifin Carprieve Novox Vetprofen Non steroidal Nonsteroidal Non-steroidal NSAID NSAIDs	Clinical improvement

In veterinary medicine, the patient group makes up two different sets of terms: the species, and the condition of interest. The acronym SPICO has been suggested for veterinary medicine (32), starting with “Species” before “Patient group.” Note the separate search terms for plurals (e.g. dog, dogs), synonyms (e.g. osteoarthritis, arthritis, degenerative joint disease), and acronyms (e.g. NSAID for non-steroidal anti-inflammatory drug) in the example search terms. Other general considerations are to include “colloquial” terms (e.g. milk fever for hypocalcaemia; cherry eye for nictitans gland prolapse) and eponyms (e.g. Johne's disease for paratuberculosis). When considering synonyms, active ingredients of products (e.g. meloxicam) are the most important terms to look for, although trade names (e.g. Metacam) can also be searched. However, registered trade names differ between countries and, although they may be included as synonyms, they should not be solely relied upon.

Another technique to help with searching inclusivity is truncating or stemming a search term. This is indicated by the addition of a non-letter character, often ^*^ or $ depending on the database. Truncated terms can save time when it is likely a number of relevant terms will have the same primary structure (e.g. desex^*^ or desex$ instead of searching for desex, desexed, desexing). These symbols can also be used in the middle of terms to search for different spellings (e.g. “sterili$ation” could be used to represent both the English “sterilisation” and American “sterilization” spellings); this is termed a wild card. Consult the help documentation for each database searched for guidance.

Whilst it is important to identify outcome terms for the PICO as these will assist in determining which of the results are most appropriate, they are often not included in the search. Results from a search of the Patient, Intervention, and Comparison typically yield a sufficiently small number of results that are easily and quickly assessed. Additionally, outcomes may not be clearly defined, it may be difficult to identify all relevant terms for outcomes, and the more concepts that are combined, the greater the risk of excluding a relevant article. Being as specific as possible with the “O” or outcome in the PICO is also useful and important in the appraisal phase of evidence reviews (see section Appraisal of the Evidence).

#### Structuring the Search and the Use of Boolean Operators

Although the CAT methodology is quite structured, there is a degree of choice and flexibility in how the search is carried out, depending on the timespan available and anticipated amount of evidence. To create a search that is broad (“sensitive”) yet relevant (“specific”), terms must be combined in an appropriate way (36). Best practice is to combine search terms and their synonyms using the Boolean operators AND and OR (12, 37); this programs the online search to retrieve relevant results. As a rule, “OR” is used when combining *within* components (e.g. all the patient terms), whilst “AND” is used when combining *separate* components (e.g. patient and intervention term lists) to assure that each component is present in the search results. Capitalizing “OR” and “AND” to denote them as search commands is best practice because it can affect the results returned in some search interfaces.

An additional consideration centres on the differing opinions as to whether the intervention and comparator components should be combined using the Boolean “OR” term. This permits citations to be identified if only one of the two components are mentioned in the abstract. Information specialists, or librarians, have specialist training and are highly skilled in generating searches that optimize the chances of identifying all relevant publications. It is best practice to seek guidance from them whether for training to conduct your own searches, or as collaborators.

The search strategy for the above scenario might appear as follows:

(dog OR dogs OR canine OR canines OR Canis)
AND
(osteoarthritis OR osteoarthritic OR OA OR arthritis OR arthritic OR joint disease OR joint diseases OR Degenerative Joint Disease OR DJD)
AND
(meloxicam OR Loxicom OR Metacam OR Inflacam OR Rheumocam OR Meloxidyl) OR (carprofen OR Rimadyl OR Canidryl OR Carprodyl OR Rimifin OR Carprieve OR Novox OR Vetprofen) OR (non steroidal$ OR nonsteroidal$ OR non-steroidal$ OR NSAID$)

**Figure 4:**
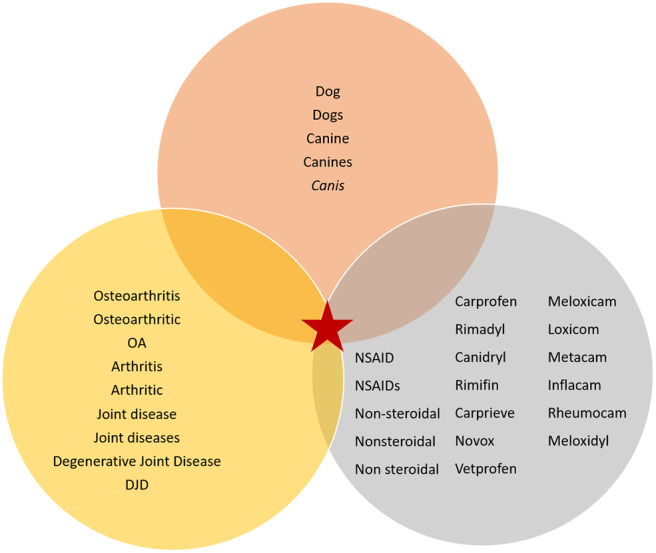
Venn diagram illustrating the interaction between the different search terms within the search components for the example scenario (denoted by a red star).

Use of AND allows papers to be identified that contain terms from all components of the search, identifying the most relevant citations, as can be seen in Figure 4. In the results, the following must be present: any term from species, any term from patient, any term from intervention, any term from the comparator.

#### Literature Databases

Once a search strategy has been created, searching can commence within a literature or bibliographic database. These differ from searching the internet using a search engine (e.g. Google or Google Scholar) in two important ways. Bibliographic databases contain journal articles that are not generally available online or accessible via internet search engines. Coverage by internet search engines is not transparent and changes frequently.

A number of bibliographic databases exist. Research suggests at least two databases should be searched, including CAB Abstracts since it contains the most comprehensive database for veterinary topics (38). The data in CAB Abstracts are available for subscription by institutions (39), and for individual subscription as VetMed Resource (40) which includes bibliographic records, limited full-text, and links to free and subscription articles. PubMed, from the US National Library of Medicine, is a freely available bibliographic database that covers biomedical sciences including a core of veterinary medicine information (41). It includes the MEDLINE database and additional bibliographic records and links to free and subscription articles.

For those employed at a university or corporation, check with your information specialists or librarians to find the databases available to you. For those not affiliated with an institution, collaboration with individuals at universities or obtaining practice or individual subscriptions to databases [e.g. VetMed Resource (40)] is useful. Some professional bodies offer access to databases as a member benefit. It would be pertinent for veterinary professionals to consider membership to relevant initiatives such as the RCVS Knowledge Library (42) which offers training in literature searching. Other cost-effective options are available (37).

#### Searching Using Database Specific Subject Headings

Search results may be improved by the inclusion of standardized terms in the search (43). These terms are specific to each bibliographic database. The content of each publication being indexed is identified, assessed, and assigned a standardized database specific term, often called a subject heading. These are “umbrella” terms for a given concept and are organized in a database-specific thesaurus. PubMed and MEDLINE use MeSH (Medical Subject Headings), CAB Abstracts uses the CABI Thesaurus. Consult the help documentation provided by the databases for guidance. Not all CAT guidance recommends the use of subject heading searches (11, 12) but when used, it is likely to improve the sensitivity of searching strategies (43) and therefore should be carried out if possible. For further information about subject headings, see the box entitled “General references about using subject headings.”

**General references about using subject headings**

EBVM Toolkit 2 (https://knowledge.rcvs.org.uk/document-library/ebvm-toolkit-2-finding-the-best-available-evidence/)

EBVM Learning Acquire (http://www.ebvmlearning.org/acquire/)

PubMed for Veterinarians (https://www.tamucet.org/product/pubmed-for-veterinarians/)

### 3. Identification of Relevant Studies

After the search has been carried out, the next step is to identify publications that can be used to answer the CAT question. Firstly, the citation must be relevant to the PICO question—it must contain all the components, including the outcome of interest. This assessment begins by looking at the title of each citation. If the title does not sound relevant, the citation is excluded and the next one is assessed (37). If the title is potentially relevant, the abstract is assessed for further detail. If the abstract is relevant, the full text of the article is scanned. If the full text article is not available, the citation may be excluded or further work undertaken to obtain a complete copy (37). There is a flow diagram that appears in White and Larson (44) that can help to facilitate the process described above.

Secondly, exclusions might apply to ensure the citations are as evidence-based as possible. For example, those citations that are not peer-reviewed (e.g. conference proceedings, textbooks, theses), do not contain evidence of research methodology (e.g. narrative reviews), or are carried out in a non-applied setting (e.g. *in vitro* research) may be excluded (36). Often if the full text version of a paper is in a language in which the authors are not sufficiently fluent, it is excluded due to the lack of time for translations in the rapid CAT process.

It is possible that at the end of this stage, no relevant peer-reviewed citations are found, or the material found provides insufficient confidence that the findings are valid. The searching strategy could be amended (e.g. using “OR” between the I and C components instead of “AND”) to “widen the net.” If this is not successful, the process of a traditional CAT ends here. Some published CATs include searches that don't return any citations to demonstrate evidence gaps (45). If the search retrieves no results but clinical decisions need to be made about a case, other forms of evidence such as conference proceedings, textbooks, narrative reviews and expert opinion could be used instead (46). Publications looking at the PICO topic as it relates to other species (including humans), or those containing *in vitro* studies could be investigated.

In the example scenario above, a MEDLINE search returned 345 citations, one of which was relevant. No papers were excluded because they were not in English, 11 papers were excluded as they were narrative reviews, conference proceedings or related to *in vitro* research, and 333 were excluded because they did not meet all components of the PICO question. A CAB Abstracts search returned 412 citations, one of which was relevant (the same paper as in the MEDLINE search). One paper was excluded as it was not in English, nine as they were narrative reviews, conference proceedings or related to *in vitro* research, and 401 were excluded because they did not meet all components of the PICO question. This left a total of one relevant paper from the two database searches, Moreau et al. (47).

### 4. Appraisal of the Evidence

One of the most important parts of the CAT process is the appraisal of the evidence. This assesses the study design and its execution (48). Often there is an assumption by medical and veterinary professionals that if something is published in a scientific journal, it is automatically valid and high quality. However, the publishing and peer review process is not flawless (49–51) and not all published articles are of equal quality (52). Therefore, it is important that all publications undergo an assessment of how they were conducted.

**Figure 5:**
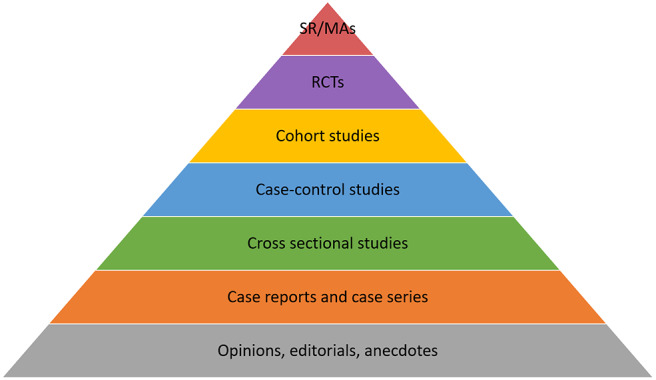
Pyramid of evidence, modified from Phillips (53). SR, Systematic reviews; MA, Meta-analysis; RCTs, Randomised controlled trials.

The first step in this process is to identify the study design and assess its place in the evidence “hierarchy” (24). All study designs have a degree of bias associated with them, but some are considered more objective than others (8). A number of schematics rank the study designs according to their inherent level of bias (hence “hierarchy”) in a “pyramid” [(2); Figure 5] or “staircase” of evidence (52). Study designs at the top of the pyramid are theoretically the least biased (e.g. systematic reviews and meta-analyses), with bias increasing toward the bottom (e.g. personal anecdotes) (24). The pyramid shape also indicates that the majority of evidence sources are at the bottom, with fewer, less biased studies at the tip of the pyramid (54).

However, the pyramid can be followed too strictly, ignoring the point that the “ideal” study design to answer a specific question relates to the type of question that is being asked.

Concerns raised include whether a case-control design is of lesser evidentiary value than cohort studies and whether the terms cohort, case control, and case series can be used to “filter” out studies of lower evidentiary value (55). Additionally, this common version of the pyramid is geared toward questions of treatment comparisons (SRs, meta-analyses and randomized controlled trials appearing at the top of the pyramid). Where a question relates to other types of clinical question, such as establishing disease prevalence, the “hierarchy” here no longer applies—for example, cross sectional studies are more appropriate to conduct in this case than randomized controlled trials (56). Additionally, there are an increasing number of qualitative research studies being undertaken in veterinary medicine; it is difficult to know where to integrate these studies into the traditional pyramid hierarchy.

It can be difficult to determine what type of study design has been carried out; the stated study design may not be correct (57, 58), which can leave CAT authors uncertain as to how to approach reading the paper. There are a number of resources that contain a good description of common study designs (1, 8, 59), including some with flow diagrams for helping to determine what type of study design has been used (8, 60, 61).

The second step in the process is to determine whether the study design has been executed in the appropriate manner; this assessment is termed “critical appraisal” (48). This is often undertaken using structured worksheets which contain questions tailored to the specific study design (13). There are many different resources that can be used for this process, but all are fundamentally similar in the questions they address. Some examples from the medical and veterinary field are highlighted in the box entitled “General references for appraising the evidence.”

**General references for appraising the evidence**

Veterinary—

CEVM website (https://www.nottingham.ac.uk/cevm/evidence-synthesis/resources.aspx)

EBVM toolkit, RCVS Knowledge (https://knowledge.rcvs.org.uk/evidence-based-veterinary-medicine/ebvm-toolkit/)

Dean RS. How to read a paper and appraise the evidence. *Practice*. (2013) 35:282–5. doi: 10.1136/inp.f1760

Downes MJ, Brennan ML, Williams HC, Dean RS. Development of a critical appraisal tool to assess the quality of cross-sectional studies (AXIS). *BMJ Open*. (2016) 6:e011458. doi: 10.1136/bmjopen-2016-011458

Moberly HK. How to read and appraise veterinary articles. *Texas Vet*. (2019) 81:54. uri: 1969.1/178285

Pinchbeck GL, Archer DC. How to critically appraise a paper. *Equine Vet Educ*. (2020) 32:104–9. doi: 10.1111/eve.12896

Medicine—

Centre for Evidence Based Medicine (https://www.cebm.net/2014/06/critical-appraisal/)

CASP (https://casp-uk.net/casp-tools-checklists/)

Joanna Briggs Institute (https://joannabriggs.org/ebp/critical_appraisal_tools)

How to read a paper series, British Medical Journal (https://www.bmj.com/about-bmj/resources-readers/publications/how-read-paper)

Crombie IK. *The Pocket Guide to Critical Appraisal*. London: BMJ Publishing Group (2009).

Greenhalgh T. *How to Read a Paper: the Basics of Evidence-Based Medicine*. 5th ed. Chichester, UK: Wiley-Blackwell (2014).

While specific questions need to be answered based on the study's design, there are key, easy questions that should be asked of all study types. These are (62):

- Does this study address a clearly focused question?
- Did the study use valid methods to address this question?
- Are the valid results of this study important?
- Are these valid, important results applicable to my patient or population?

The Centre for Evidence-Based Medicine takes a time-efficient approach to the answers to these questions, saying that if the answer is no to any of them, clinicians should avoid reading the rest of the paper as it is not relevant (62).

Veterinary professionals can worry that appraisal will be too difficult and may need advanced understanding of statistics. In reality, critical appraisal relies on the application of common sense in conjunction with an appraisal template with much of the focus on the study design, not the statistics. For example, of the 27 questions posed in the randomised controlled trial (RCT) critical appraisal sheet developed by the Centre for Evidence-based Veterinary Medicine (CEVM), only four relate to statistical calculations (63). None require the appraiser to carry out any statistical tests. There are a number of easy to understand statistical reference guides that could assist professionals to interpret common types of analyses (64–66) that could be used alongside the structured worksheets to assist them in interpreting the study results. Alternatively, assistance or further training could be sought (2, 67), but this level of higher knowledge is rarely required. Therefore, veterinary professionals should be able to appraise the vast majority of important features within each study in order to draw meaningful conclusions. If the study is well-conducted and well-reported, it should be easy to critically appraise.

In the given scenario, the paper that was identified (47) was a randomised controlled trial. After appraisal using the RCT critical appraisal sheet from the CEVM (63), the main points to note in relation to the study were:

- Prior to the study commencing:
  ∘ There was no assessment of how many animals would be required prior to the study commencing (e.g. no sample size or power calculations were presented).
- Once the study had commenced:
  ∘ The study focused on dogs weighing more than 20 kg and were older than 18 months of age with radiographic evidence of osteoarthritis in a range of joints. Subjects were excluded if there was history of other types of musculoskeletal comorbidities.
  ∘ Outcomes measured were owner activity and pain scores, clinician orthopedic examination score, ground reaction force gait analysis and biochemical, haematological and faecal assessments.
  ∘ Baseline characteristics and clinical characteristics of the subjects were not reported.
  ∘ Aggregated results were reported for most but not all parameters; it was difficult to determine basic results as a consequence.
  ∘ There were no statistically significant improvements in owner score compared to pre-treatment scores. The exception was a subset of dogs with stifle disease in the Metacam group (*n* = 6) who showed an improvement at day 30 only (not at day 60). There was no statistically significant difference between the two treatments for this measure.
  ∘ Within each treatment group, there were statistically significant improvements in clinician score (at day 30 only), and in selected ground reaction force measures compared to pre-treatment scores. There was no statistically significant difference between the performance of the two treatments.

### 5. Summarise Findings and “Clinical Bottom Line”

The last part of the process is an overall assessment of all the evidence appraised. There is no standard way of amalgamating results from appraisals in the CAT format (36) but it becomes easier with practice. Challenges include comparisons of different types of study design (e.g. a randomized controlled trial and cohort study), and where different studies report conflicting answers to the question. Conflicting answers could be related to the varying abilities/characteristics and biases inherent in the different study types (35), or because different populations have been studied (e.g. shelter animals vs. owned animals; a study based in Australia vs. a study based in Canada). This is where the judgement of the veterinary professional becomes important. It is a common occurrence that, even after reading the evidence, there is no clear, definitive conclusion. It should be noted that such an outcome is distinct from a conclusion stating that there is no effect of the intervention.

In the given scenario, the study weaknesses were felt to be substantial enough to conclude it was not possible to answer the clinical question. The clinical bottom line was that there was insufficient evidence to demonstrate a difference in relation to the greatest clinical improvement between the performance of meloxicam or carprofen in dogs with osteoarthritis. For an overall summary of the example CAT provided here, refer back to Figure 3.

## Publishing a CAT

Production of the CAT can be carried out by more than one author (45) to increase objectivity and reduce bias. Once the question and search strategy are agreed, multiple authors may independently search the literature and/or, more commonly, agree on any relevant studies. They reach a consensus on which studies to include and then independently appraise their quality, before collaborating again to summarize the findings and arrive at the clinical bottom line. There is a lack of guidance on reporting CATs in the literature, and those that do exist tend to be journal specific. At the time of writing, the Veterinary Evidence journal had the most comprehensive guidance for reporting Knowledge Summaries (a form of CAT) (33), with minimal guidance provided by Equine Veterinary Education (68). It is recommended also to look at the examples following this section for further guidance on reporting. Journals such as the Veterinary Record (publish BestBETs—a form of CAT—and other formats), BMC Veterinary Research, Equine Veterinary Education and Veterinary Evidence (publish Knowledge Summaries) have published CATs previously.

### Good Examples of Critically Appraised Topics From Medicine and Veterinary Medicine

There are a number of excellent examples of CATs and resources available to help facilitate the construction of CATs, both in the medical and veterinary fields. This section will focus on published examples of CATs, collections of existing CATs, and website resources that can be utilized to construct CATs. The applied nature of CATs means that many of the most useful “how to” resources are not published in peer-reviewed journals, but on university webpages, open access online tutorials or online databases.

### Medicine

Over time there have been a number of medical CAT databases in existence; in 2005 there were at least 13 different places where medical CATs appeared (69); it is unknown how many of these are still regularly contributed to. Software was developed to be able to search simultaneously across a number of different CAT databases [“CAT crawler”; (70)], but widespread use of this is not evident in the literature.

A good example of a working database of CATs is BestBETs (www.bestbets.org). This database was constructed by emergency clinicians working at the Manchester Royal Infirmary in the UK, in response to a lack of high quality evidence for some of what was seen regularly in emergency care (71), hence the use of the term “Best Evidence Topics (BETs).” Some of these BETs are also published in peer-reviewed journals. The topics covered in this database have expanded to include other specialties besides emergency medicine, including cardiothoracics and paediatrics.

### Veterinary Medicine

There are numerous different formats of CATs available in veterinary medicine, most of which have emerged over the past 10 years. There are some differences between these formats in relation to how the review question has come about, what format the review is available in (e.g. on a website, published literature), and how the “review” component of each format occurs (e.g. number of authors, reviewers etc.), but they essentially follow the same process. The advantage for veterinary professionals is that there are several CAT collections available to utilise for decision making in clinical practice. The collections of veterinary CATs available at the time of article preparation are listed alphabetically in Table 2. The majority of these are freely available, although not all appear to be current and are being updated at variable frequencies. Published examples of CATs and useful web sources to help create CATs can be seen in the inset boxes below.

**Table 2:** Collections of veterinary CATs.

**Name of CAT collection**	**References**	**Start date of CATs**	**Date of last CAT**	**Approach**	**Type of source**	**Frequency of updates**
Banfield Applied Research and Knowledge (BARK) CATs website	https://www.banfield.com/veterinary-professionals/resources/research/cats	Nov 2009	2013	Single author reviews	Free to view	Unknown
BestBETs for Vets website (and a selection of these in the Veterinary Record journal)	http://bestbetsforvets.org/	Sept 2013	Current at time CAT review article published	Multi-author reviews	Free to view	Every 2 years
Equine Veterinary Education journal	https://beva.onlinelibrary.wiley.com/hub/journal/20423292/homepage/critically_appraised_topics_for_clinical_evidence_in_equine_practice.html	March 2015 (for clinical evidence series of CATs)	2020	Single author reviews	Free to view	No set timeline
Veterinary Evidence journal (called Knowledge Summaries)	https://veterinaryevidence.org/index.php/ve/index	Oct 2015	Current at time CAT review article published	Single author reviews	Free to view	Most popular automatically updated every 2 years; others when required
Veterinary Prescriber website	https://www.veterinaryprescriber.org/	Mar 2014	Current at time CAT review article published	Multi-author reviews	Subscription based	Variable; 3 years or more
“Where's the evidence” series in the Journal of the American Veterinary Medical Association in conjunction with the Evidence Based Veterinary Medicine Association	https://avmajournals.avma.org/loi/javma/	Nov 2009	Aug 2011	Multi-author reviews	Subscription based	Unknown

**Published examples of veterinary CATs:**

There are several good examples of veterinary CATs that have been published in the literature. Two can be seen here, both of which are free to view. These examples demonstrate a contrast in relation to the types of question and approaches that can be used under a CAT format.

Finka LR, Ellis SLH, Stavisky J. A critically appraised topic (CAT) to compare the effects of single and multi-cat housing on physiological and behavioral measures of stress in domestic cats in confined environments. *BMC Vet Res*. (2014) 10:73. doi: 10.1186/1746-6148-10-73

This CAT contributed to the development of welfare guidelines for unowned cats (72).

Olivry T, Mueller RS, Prelaud P. Critically appraised topic on adverse food reactions of companion animals (1): duration of elimination diets. *BMC Vet Res*. (2015) 11:3. doi: 10.1186/s12917-015-0541-3

**Useful web sources:**

Medicine:

“How to” resources—

Centre for Evidence Based Medicine CATmaker: (https://www.cebm.net/2014/06/catmaker-ebm-calculators/)

Physiopedia: (https://www.physio-pedia.com/Critically_Appraised_Topics)

Veterinary medicine:

Other CATs—

Healthy Feet website: (https://www.cattle-lameness.org.uk/critically-appraised-topics/)

BMC adverse food reaction CATs: (https://www.biomedcentral.com/collections/catsfoodreactions)

## Some of the Limitations and Misinterpretations Associated With Cats

The main limitation associated with the use of the CAT methodology is the “quick and dirty” nature of the process. Due to the rapid approach, the process is not as detailed nor in depth as other types of review, and therefore there is more potential to miss relevant evidence sources (9). This may mean that a CAT may not be representative of the totality of the evidence in existence on a particular topic (36). In addition, the questions asked when using a CAT format are usually narrow and tend to be very specific to a clinical scenario or experience (9). This sometimes limits the ability to translate findings to a wide variety of situations. However, there are so few evidence-based resources and limited funding for CAT resources for veterinary professionals that any structured reviews can be of benefit to clinical decision making. As with any type of review publication, they can become outdated and should be re-assessed regularly (9).

There are other pitfalls associated with this methodology which are inherently related to structured reviews generally. If only “colloquial” terms are used (those used locally or regionally) to describe diseases/conditions/procedures then it is more likely a CAT author from a different part of the world may miss a relevant publication. For example, the term “tup” can be used to describe a male sheep in the UK; in other countries this term is not generally used. The majority of known CAT collections in veterinary medicine are published in English, and to the authors' knowledge, none of the reviews in these databases go to the extent of searching for non-English publications for inclusion. This is a distinct limitation (73) but is also likely to be related to the rapid nature of these reviews in relation to the delay it may take for additional searching and translation to be undertaken.

For relevant studies to be identified, published research must be indexed correctly. Information specialists rely on authors identifying the most appropriate key words for their publication and ensuring the most important terms are included in the title and abstract. It also depends on the terminology used to describe disease conditions or procedures. Additionally, depending on the database in question, some of the indexing of veterinary related publications is done by personnel who may not necessarily be familiar with some of the conditions that afflict animals. Automated indexing systems can both omit relevant subject headings from a record which can impact on retrieval or include erroneous subject headings. All of the above can impact on whether specific publications are returned after a structured search has been performed.

There are sometimes misconceptions by veterinary professionals in relation to these clinically relevant reviews of the literature, analogous to those held by some medical professionals in relation to clinical guidelines (74). They can be seen as the definitive answer, from which health professionals are only allowed to deviate for good reasons. This can be comforting, particularly to those inexperienced in clinical decision making, such as new graduates. Alternatively, CATs can be regarded as over-prescriptive, too restrictive in scope and even draconian. However, these reviews should always be applied contextually to the patient in front of the decision-maker. If the study populations are substantially different to their own patients, the veterinary professional may deem the CAT irrelevant and choose to ignore it (24). The evidence must be applied within the context of the circumstances of the patient and owner in order for the clinical plan and treatment to have the greatest chance of success.

## Further Developments

Excluding for educational purposes, the role of the CAT appears to have been superseded by SRs, which are often used as the basis for clinical guidelines for medical practitioners [e.g. in the UK, Clinical Knowledge Summaries; (75)]. SRs are a more thorough representation of the existing evidence than CATs, and include both published and, often, unpublished sources of information. For areas not covered by such guidelines (e.g. common questions still to be answered, and areas where it is inherently difficult to undertake unbiased types of study such as randomised controlled trials, for example in emergency medicine), CATs will continue to play an important role in clinical practice. In veterinary medicine, there are unlikely to be large numbers of systematic reviews generated in order to develop clinical guidelines, primarily due to a lack of both suitable research funding and appropriate skills within the veterinary profession. However, many veterinary and nursing undergraduate courses and further education courses for technicians globally include elements of EBVM training (such as how to carry out a CAT) within them (27, 28) and there are also opportunities now for post-registration training and continuing professional development in these skills (32, 67, 76, 77). This suggests that the skill base may well increase in the future. With some additional work, the CATs undertaken by students that are currently kept internally within institutions could become publicly available CAT collections. Alternatively, with some assistance from educators, student CATs could be published in veterinary journals. There are awards available for students to publish CATs currently (78), which should facilitate this process.

With the creation of more CAT collections in the veterinary sphere (Table 2), professionals can use CATs without requiring the same skills needed to generate them. To facilitate carrying out the CAT process in clinical practice, adequate time must be given to professionals to be able to perform searches and interpret evidence during their working day. This is a bigger challenge for the profession that must be prioritized moving forwards. With the rise of corporate practice groups, there have emerged roles with the responsibility of ensuring EBVM-based practice. This may accelerate the prioritization of evidence reviewing as part of a veterinary professionals' role, which could increase the demand for CATs and thereby facilitate formation of a more centralized source for professionals.

For busy practitioners, having numerous different CAT collections to search across is suboptimal. In the future it may be that provision of software, such as the “CAT crawler” (70) would overcome this barrier. However, this requires funding for development and maintenance which, for these sorts of resources, is unlikely to be prioritized by funding bodies. Additionally, in order to increase the translatability of the CATs in these collections, adding a patient perspective section may add a different dimension. The CAT undertaken by Wootton et al. (79) that appeared in the British Journal of Dermatology includes such a section, so a template is already in existence that could be utilized. A similarly motivated patient perspective column has recently been initiated as a feature in the Veterinary Record journal (80) which demonstrates the power of the client's voice.

## Conclusion

The CAT framework is still a current and useful process for veterinary professionals to use primarily for evidence-based clinical decision making and for undergraduate and post-registration training. With the provision of new CAT collections that can be utilized often at no cost, there are good options available for those in clinical practice who do not yet have the skills to generate CATs themselves. All veterinary professionals, with regular practice, have the ability to successfully navigate the CAT process. However, time must be given to those in clinical practice for the development of these skills so that more CATs can be generated, facilitating excellent evidence-based care of clients and their animals.

## Author Contributions

MB, LC, HD, and LM were involved in creating the framework for the manuscript. MB, SA, ZB, LB, LC, HD, VF, DG, HM, LM, JS, and CW contributed to acquisition of data (publications) for the work. MB wrote the draft manuscript. MB, SA, ZB, LB, LC, HD, VF, DG, HM, LM, JS, and CW contributed to editing the manuscript, read and approved the final manuscript.

## Conflict of Interest

The authors declare that the research was conducted in the absence of any commercial or financial relationships that could be construed as a potential conflict of interest.
